# Sleep, Health Care–Seeking Behaviors, and Perceptions Associated With the Use of Sleep Wearables in Canada: Results From a Nationally Representative Survey

**DOI:** 10.2196/68816

**Published:** 2025-06-17

**Authors:** Karianne Dion, Meggan Porteous, Tetyana Kendzerska, Ashley Nixon, Elliott Lee, Massimiliano de Zambotti, Sheila N Garland, Mandeep Singh, Gino De Luca, Samuel Gillman, Andrée-Ann Baril, Dave Gallson, Rebecca Robillard, Canadian Sleep Research Consortium

**Affiliations:** 1 Sleep Research Unit, University of Ottawa Institute of Mental Health Research at The Royal, 1145 Carling Avenue, Ottawa, ON, Canada, 1 6137226521; 2 School of Psychology, University of Ottawa, Ottawa, ON, Canada; 3 Department of Medicine, Faculty of Medicine, The Ottawa Hospital Research Institute, University of Ottawa, Ottawa, ON, Canada; 4 Center for Health Sciences, SRI International, Menlo Park, CA, United States; 5 Department of Psychology, Memorial University, St-John's, NL, Canada; 6 Toronto Sleep and Pulmonary Centre, Toronto, ON, Canada; 7 Department of Anesthesiology and Pain Management, Women’s College Hospital, Toronto, ON, Canada; 8 National Research Council Canada, Ottawa, ON, Canada; 9 Sleep, Cognition, & Neuroimaging Lab, Montréal, QC, Canada; 10 Department of Medicine, Université de Montréal, Montréal, QC, Canada; 11 Center for Advanced Research in Sleep Medicine Research Center of the CIUSSS-NIM, Hôpital du Sacré-Coeur de Montréal, Montréal, QC, Canada; 12 Mood Disorders Society of Canada, Belleville, ON, Canada

**Keywords:** sleep wearables, sleep tracking, sleep monitoring, anxiety, mental health, population, Canada

## Abstract

**Background:**

The popularity of sleep-tracking wearables has surged worldwide. Yet, there are significant gaps in understanding the real-life implications of this phenomenon. While wearables may offer insights about sleep and promote sleep health awareness, evidence remains mixed on whether they lead to improved sleep outcomes or fuel sleep anxiety.

**Objective:**

This study aims to (1) determine the prevalence and sociodemographic predictors of using sleep wearables in Canada, (2) evaluate the perceived effects of wearable use on sleep and stress, (3) compare sleep and health care–seeking behaviors in users and nonusers, and (4) investigate the moderating effects of wearable use on the association between sleep and anxiety.

**Methods:**

An online survey investigating sleep and mental health was distributed to a representative sample of 1200 Canadians. The survey included questions on demographics, wearables use, sleep patterns, health care–seeking behaviors, insomnia (ISI-3 [Insomnia Severity Index-3]), and anxiety (GAD-7 [Generalized Anxiety Disorder-7]) symptoms. Analyses relied on descriptive statistics and logistic regression (aims 1 and 2), multivariate analyses of covariance and chi-squared analyses (aim 3), and multiple regression (aim 4).

**Results:**

Among the 1200 respondents (n=636, 53% female; aged 16 to 88 years), 19.3% (n=231) reported using a wearable device to monitor sleep. Several sociodemographic variables were associated with an increased likelihood of using wearables including: youth, being retired, being part of a racialized minority group, earning a higher income, having greater health care coverage, having a sleep disorder, and having a mental disorder (*χ*^2^_14_=110.2, *P*<.001). Of all wearable users, nearly 45% felt that using sleep wearables had a positive effect on their sleep (n=102) and stress levels (n=97), while 4.5% (n=10) noted a negative effect. Compared to nonusers, wearable users reported 13 minutes longer sleep onset latency (*F*_1,1151_=5.21, *P*=.02, ƞp^2^=0.005), slept about 1 hour less (*F*_1,1143_=31.60, *P*<.001, ƞp^2^=0.027), and endorsed more severe insomnia symptoms (*F*_1,1119_=4.04, *P*<.05, ƞp^2^=0.004). After adjusting for the presence of sleep disorders, only the differences in sleep duration remained. The proportion of wearable users was almost twice as high in those having informed a health care provider about sleep difficulties (*χ*^2^_2_=35.4, *P*<.001) and in those having used sleep medications (*χ*^2^_3_=38.7, *P*<.001). Wearable use was identified as a moderator of the effect of anxiety symptoms on sleep duration, with wearable users showing a steeper decline in total sleep time as anxiety increased compared to nonusers (*F*_1,1165_=17.5, *P*<.001).

**Conclusions:**

One in 5 Canadians acknowledged having used sleep wearables. Predictors include younger age, higher income, and having a sleep or mental disorder. Although many individuals reported positive effects of sleep wearables, wearables use strengthened the link between short sleep and anxiety. Expanding our understanding of the factors associated with beneficial versus detrimental use of sleep wearables may help support more informed applications.

## Introduction

With growing public interest in sleep health, an increasing number of people are turning to technologies to monitor their sleep. In 2021 alone, the global market for sleep wearables was valued at 1.8 billion and is expected to reach 3.1 billion by 2028, with North America being the largest market for these devices [1]. Sleep technology is advancing rapidly, with a wide range of wearables now available to consumers, with common examples being wrist-worn devices, rings, and headbands. These devices often include multiple sensors that measure various metrics such as body movement, skin temperature, heart rate, blood oxygen levels, and even brain activity. Due to concerns regarding the accuracy and transparency of consumer-oriented wearables data [2-5], most studies to date have focused on assessing their performance and on delimiting their place in clinical and research settings [6,7]. Regardless of the varying level of evidence supporting their validity, wearables are commonly used by consumers daily. There is thus a need for naturalistic studies to investigate the real-world implications of their use.

There is an ongoing debate about the potential harms and benefits of consumer wearables for sleep. In line with the quantified-self movement (ie, self-knowledge through numbers), some studies have found the use of sleep wearables to be associated with improvements in sleep quality and quantity [8]. Although not fully understood, one of the suspected mechanisms linking wearables to better sleep is the identification of sleep-wake patterns that can provide insight about potential adjustments that could improve sleep, for example, optimal bed and wake times. For example, a longitudinal study by Kuosmanen and Kuosmanen [9] found that tracking sleep over time can help users identify factors negatively affecting sleep quality. Other proposed mechanisms include improvements in sleep hygiene or the sleep environment based on wearable feedback and reminders, increases in physical activity when wearing the device, and identification of potential sleep disorders.

Although sleep wearables may improve sleep for some people, some studies show that they can also have adverse effects. Notably, health care providers have reported a noticeable rise in sleep-related anxiety and concerns prompted by the use of sleep wearables in some patients [5,6,10]. As described by case reports, a fixation on sleep tracker data, fueled by a preoccupation to maintain high “sleep scores,” may drive some individuals to strive for the “perfect sleep,” a phenomenon that has now been coined “orthosomnia” [10]. Orthosomnia is linked to dysfunctional beliefs about sleep and has the potential to increase sleep-related anxiety as well as harmful sleep behaviors that may exacerbate insomnia symptoms, such as spending an excessive amount of time in bed awake. These sleep ideals have been described as particularly difficult to modify, raising additional concerns about the importance that certain consumers may place on sleep wearables data [10]. Kuosmanen and Kuosmanen [9] also reported that some participants experienced stress from using sleep tracking devices, highlighting the need for careful consideration in their use. Moreover, many sleep trackers emit light and vibration alerts, and some devices may also be associated with a sense of discomfort, which may directly disturb sleep [4]. Given these potential drawbacks, it becomes essential to explore the broader implications of wearable technology.

To date, very few studies on wearable trackers have been conducted in population-based samples, which considerably limits the generalization of available evidence on their implications for sleep, health care, and mental health. Results from a national survey investigating the self-tracking of physical activity in Canada showed that 1 in 4 Canadians reported using a wearable or smart medical device, and over half of those engaged in self-tracking regularly [11]. Groups who were identified as more likely to engage in self-tracking included young and middle-aged adults (aged 18‐34 years), and those who were healthy, employed, had a university education, and had an annual family income of over CAD $80,000 (US $63,600). Respondents in this study endorsed monitoring their physical activity, nutrition, and sleep patterns as the main reasons for engaging in self-tracking, and over 65% reported that this had allowed them to be better informed of their general health (n=387/580, 66.6%) and to maintain or improve their health condition (n=398/580, 68.5%).

Given the elevated prevalence of comorbid medical and sleep disorders in clinical populations [12,13], there is an important need to study the use of wearables in samples with mental health difficulties. Notably, a survey conducted in a representative sample of Americans with histories of psychological disorders found that being female, having a college-level degree, being younger, and earning a higher income were associated with a higher likelihood of using wearable devices [14]. In this previous study, about 28.1% (n=320/1139) of individuals with a history of anxiety or depressive disorder reported using a wearable device, which was comparable to the rates reported by the general population. Despite these insights, there remain important gaps in understanding the real-life implications of using wearable devices, especially regarding sleep, sleep-related health care behaviors, and mental health in a North American context.

The objectives of our study were to (1) estimate the proportion of Canadian adolescents and adults who have used sleep wearables and identify sociodemographic predictors associated with the use of sleep wearables, (2) evaluate the perceived effects of using sleep wearables on sleep and stress levels, (3) compare the sleep profiles and sleep-related health care of wearable users and nonusers, and (4) investigate whether the use of wearables moderates the effects of anxiety symptoms on sleep duration.

## Methods

### Study Design

Data for this study were collected as part of a larger national sleep and mental health survey co-developed by the Mood Disorders Society of Canada, a nongovernmental organization advocating for mental health, as well as scientists and clinicians from the Canadian Sleep Research Consortium and the Canadian Sleep Society.

The survey was designed through a series of collaborative meetings with representatives of the 3 organizations, who reviewed relevant literature and reached consensus on the survey content. The full survey consisted of a total of 68 items, including validated questionnaires as well as custom-built questions about sleep, mental health, wearables use, sleep interventions, health care services, and sociodemographic characteristics. It was reported following guidelines from the Checklist for Reporting Results of Internet E-Surveys (Checklist 1) [15]. The survey was based on a decisional tree structure to personalize subsets of questions for each participant. It was available in both French and English and it took approximately 20 minutes to complete the full survey. Participants could choose to leave any question unanswered at their discretion and could leave and return later to complete the survey at their convenience. For the current report, relevant items were selected to address the objectives of this specific subproject.

### Ethical Considerations

This project was approved by the Research Ethics Board of the Royal Health Care Group (REB#2021038). Informed consent was obtained on the first page of the survey, and confirmed that participants were taking part in this study voluntarily. It also informed participants about the length of time of the survey, where and for how long data was stored, who the investigators were, and the purpose of this study. All data collected were anonymous. Respondents who completed the survey received compensation for their time. As per their standard processes, Dynata provided an equivalent level of reward for all respondents based on the amount of effort required.

### Recruitment and Data Collection

Narrative Research, a subcontracted recruitment firm, assisted with the programming of the survey and its beta-testing, data collection, and creation of the initial database. The survey was nested in Voxco, a secure online platform with servers located in Canada. Narrative Research subcontracted Dynata (ie, a global provider of data and insight services specializing in survey research and audience data) for the recruitment of a representative sample of Canadians. Artificial intelligence tools were used to send out survey invitations to a random sample of Dynata members. Invitations to complete the survey were sent directly to Dynata members via an online dashboard. Each invitation included a unique link to the online survey hosted by Voxco. Hence, this was a closed survey. The survey never displayed a second time once the user had completed it. The online survey was distributed between September 21 and 24, 2021.

Inclusion criteria were to be a Canadian resident and to be aged 16 years or older. A sample size of 1200 participants was chosen to approach a sufficient level of generalizability of findings to the broader Canadian population. The sample was selected semirandomly based on age, sex, and geographic region to compose a nationwide representative sample.

### Measures

Custom-made questions assessed general sociodemographic factors, sleep wearable use, and health care–seeking behaviors. Specific questions about the use of sleep wearables included “Have you ever used a wearable device to monitor your sleep (eg, Fitbit, Apple watch, Oura ring)?” (Fitbit [Google LLC], Apple watch [Apple Inc], Oura ring [Ouraring Inc]) and, if the first question was answered “yes,” “What impact did using a wearable device to monitor your sleep have on your: (a) sleep; (b) stress level?” Choice responses related to the effects of wearable use ranged from “negative,” “no impact,” “somewhat positive,” “positive,” “very positive” to “varied across time.” As space was limited, selected items from the Pittsburgh Sleep Quality Index (PSQI) [16] were adapted to assess sleep quantity over the past 2 weeks (ie, first 4 items of the PSQI). Participants were also asked to report whether they had a current diagnosis of a sleep disorder given by a health care professional (yes or no). Symptoms of insomnia and anxiety experienced over the past 2 weeks were evaluated via the brief version of the ISI-3 (Insomnia Severity Index-3) [17] and the GAD-7 (Generalized Anxiety Disorder-7) [18], with higher scores indicating more severe symptoms.

### Statistical Analysis

Analyses were conducted on all available data for each given variable. Descriptive statistics were used to determine the frequency of wearable use among respondents. A logistic regression was used to assess which sociodemographic factors independently explained the use of wearables. The predictors included in the model were age (continuous), sex (female vs male), race or ethnicity (due to limited sample sizes, all individuals from minority groups were combined into 1 group and compared to a group combining White-North American and White-European people; exploratory analyses for each racial or ethnic groups are reported in Table 1), occupational status (retired, student, or unemployed vs employed), level of education (high school or less vs college/CEGEP [collège d'enseignement général et professionnel] vs university), income (low [total household income <CAD $33,000] vs >CAD $33,000, a currency exchange rate of CAD $1=US $0.8 was applicable]), insurance coverage (private or company plan, both provincial and private care plan, or neither provincial nor private vs provincial health care), sleep disorder diagnosis (yes vs no), and mental health diagnosis (yes vs no). Age, sex, occupational status, level of education, and income were integrated as predictors in the model to replicate findings from previous studies [11,14] to a current Canadian context. Other variables such as race or ethnicity, insurance coverage, sleep disorders, and mental health diagnosis were also included in the model to explore potential disparities in wearable use based on systemic and health-related factors that may influence access, need, and engagement with sleep tracking technology. There was no evidence of collinearity, as evidenced by variation inflation factors and condition indices.

**Table 1.** Sleep wearables use stratified by self-identified racial or ethnic groups. A total of 92 respondents self-identified as belonging to multiple racial or ethnic groups.

Racial or ethnic group	Values, n (%)
Black or Black African heritage	4 (2.4)
East Asian (eg, Chinese, Japanese, or Korean)	18 (11.3)
Indigenous (First Nations)	11 (6.8)
Indigenous (Inuit)	0 (0)
Indigenous (Métis)	2 (1.1)
Latin American (eg, Argentinian, Chilean, or Salvadorian)	2 (1.1)
Middle Eastern or West Asian (eg, Lebanese, Iranian, or Afghan)	2 (1.4)
South Asian (eg, Indian, Pakistani, Punjabi, or Sri Lankan)	15 (9.6)
Southeast Asian (eg, Filipino, Malaysian, Vietnamese, Cambodian, or Laotian)	3 (1.7)
White-European (eg, English, Italian, Portuguese, or Russian)	36 (23.4)
White-North American (eg, Canadian or American)	74 (47.6)
Other	4 (2.5)

Frequency descriptive statistics were used to characterize the perceived effects of wearable use on sleep and stress. Multivariate analyses of covariance were conducted to compare self-reported sleep onset latency, sleep duration, and ISI-3 total scores in wearable users and nonusers while adjusting for age, sex, ethnicity, occupational status, level of education, income, and insurance coverage. The same model was replicated with the addition of sleep disorder diagnosis as a covariate. Chi-squared analyses compared the proportions of wearable users and nonusers who reported having used sleep medications and having informed a health care provider about sleep difficulties.

Lastly, a hierarchical multiple regression, with sleep duration as the dependent variable, assessed the increase in variation explained by the addition of an interaction term between the severity of anxiety symptoms (GAD-7) and wearable usage to a main effects model. This analysis was adjusted for the following covariates: age, sex, ethnicity, occupational status, level of education, income, and insurance coverage. Linearity was established by visual inspection of a scatterplot and there was no evidence of multicollinearity, as evidenced by tolerance values. Visual inspection of the histogram suggested a normal distribution of the studentized residuals. Secondary moderation analyses with the same model were conducted in the subgroup without any self-reported sleep disorders.

For chi-squared analyses, Cramer V was used as an effect size, with 0.10, 0.30, and 0.50 as the thresholds for small, medium, and large effect sizes, respectively [19]. For all other analyses, partial eta-squared (ƞp^2^) were used to determine effect sizes with the following thresholds: >0.02 (small), >0.13 (medium) and >0.26 (large) [20]. All analyses were conducted in IBM SPSS Statistics for Windows (version 27; IBM Corp).

## Results

### Sample Characteristics

A total of 1200 individuals aged between 16 to 88 years completed the survey (see Table 2 for full sample characteristics). In brief, 53% (n=636) of the sample were females, 95.1% (n=1141) had completed high school or postsecondary education, 44% (n=528) had full-time employment, and 27.7% (n=332) were retired. Among all respondents, 19.9% (n=238) reported currently having a diagnosed sleep disorder. Overall, the distribution of respondents across provinces and the main demographic characteristics were comparable to Canadian Census data [21-23].

**Table 2.** Global sample characteristics in relation to 2021 Canadian Census data [21-23].

		Values, n (%)	Canadian population, % [21-23]
Age		47 (30)^a^	41.9
Sex			
	Females (versus males)	636 (53)	50.9
	Prefer not to say	1 (0.1)	—^b^
Gender			—
	Boy or man	552 (46)	
	Girl or woman	633 (52.8)	
	Transgender (female to male)	4 (0.3)	
	Transgender (male to female)	1 (0.1)	
	Gender fluid	3 (0.2)	
	Gender nonbinary	3 (0.3)	
	Prefer not to say	5 (0.4)	
	Other (2-spirited, neko trans, per or peo)	4 (0.4)	
Ethnicity			—
	Black or Black African	34 (2.8)	
	East Asian	79 (6.6)	
	Indigenous (First Nations)	39 (3.2)	
	Indigenous (Inuit)	14 (1.2)	
	Indigenous (Métis)	9 (0.7)	
	Latin American	10 (0.9)	
	Middle Eastern or West Asian	19 (1.6)	
	South Asian	59 (4.9)	
	White-European	318 (26.5)	
	White-North American	660 (55)	
	Prefer not to say	26 (2.1)	
BMI		25 (7)^a^	—
Province			—
	Alberta	142 (11.8)	11.3
	British Columbia	126 (10.5)	13.3
	Manitoba	60 (5)	3.6
	New Brunswick	28 (2.4)	2
	Newfoundland and Labrador	22 (1.8)	1.3
	Nova Scotia	42 (3.5)	2.5
	Ontario	473 (39.4)	37.9
	Prince Edward Island	1 (0.1)	0.4
	Quebec	275 (22.9)	22.5
	Saskatchewan	31 (2.6)	3
Total household income (CAD $)^c^			
	<33,000 (versus >33,000)	207 (17.2)	16.4
	Prefer not to answer	93 (7.7)	—
Education level			—
	Less than high school	44 (3.6)	
	High school	278 (23.2)	
	CEGEP^d^	79 (6.6)	
	College or equivalent	278 (23.2)	
	University graduate	360 (30)	
	University postgraduate	153 (12.7)	
	Prefer not to say	9 (0.7)	
Occupational status			—
	Employed full-time	528 (44)	
	Employed part-time	106 (8.8)	
	Self-employed	63 (5.3)	
	Unemployed but looking for a job	56 (4.7)	
	Unemployed and not looking for a job	47 (3.9)	
	Student	21 (2.2)	
	Retired	332 (27.7)	
	Volunteering full time	2 (0.2)	
	Short-term disability	5 (0.4)	
	Long-term disability	44 (3.7)	
	Prefer not to answer	15 (1.2)	

a Median (IQR).

b Not available.

c A currency exchange rate of CAD $1=US $0.8 was applicable.

d CEGEP: collège d'enseignement général et professionnel.

### Sleep Wearable Use and Sociodemographic Predictors

Of 1200 responders, 19.3% (n=231) reported having ever used a wearable device to monitor sleep. Of the 9 predictor variables, all were statistically significant except sex and education level. Younger age, being retired, being part of a racial or ethnic minority group, having an income ≥CAD $33,000 (US $26,070), being on both provincial and private health care plans, having a sleep disorder, and having a mental health disorder were associated with an increased likelihood of having used a sleep wearable, while being a student was associated with a decreased likelihood of having used a wearable (Table 3). Given the insufficient statistical power to add racial minorities separately within the model, secondary descriptive analyses were conducted to stratify the use of sleep wearables across self-identified ethnic groups (Table 1). Among those who endorsed having used wearables to monitor sleep, 11.3% (n=18) identified as East Asian, 9.6% (n=15) identified as South Asian, 23.4% (n=36) identified as White-European, and 47.6% (n=74) self-identified as White-North American.

**Table 3.** Sociodemographic predictors of wearable use.

		ß	SE	EXP (B)^a^	95% CI for EXP (B)		*P* value
					Lower Bound	Upper Bound	
Age (per year)		−0.04	0.01	0.96	0.95	0.98	<.001
Sex (female vs male)		−0.12	0.17	0.89	0.64	1.25	.50
Ethnicity (racial minorities^b^ vs White)		0.61	0.19	1.84	1.26	3.66	.001
Occupational status (vs employed)							.01
	Retired	0.69	0.31	1.99	1.07	3.67	.03
	Student	−1.81	0.81	0.16	0.03	0.80	.03
	Unemployed	−0.29	0.33	0.75	0.40	1.42	.38
	Education (vs university)						.76
	College or CEGEP^c^	0.15	0.20	1.16	0.78	1.73	.46
	High school or less	0.87	0.23	1.09	0.70	1.69	.70
Income (<CAD $33,000 vs ≥CAD $33,000)^d^		−0.77	0.28	0.46	0.26	0.80	.006
Insurance coverage (vs provincial health care)							.02
	Private or company plan	0.42	0.23	1.52	0.97	2.38	.07
	Provincial and private plan	0.73	0.23	2.08	1.32	3.29	.002
	Neither provincial nor private	0.17	0.29	1.18	0.67	2.08	.56
Sleep disorder (yes vs no)		0.54	0.21	1.72	1.15	2.58	.009
Mental health disorder (yes vs no)		0.63	0.22	1.83	1.19	2.82	.006

a Exponentiated regression coefficient

b Global model: *χ*2_14_=110.2, *P*<.001; explained 15.8% (Nagelkerke R2) of the variance in wearable usage. Variation inflation factors for all predictors were <1.29, and all condition indices were <15. Due to the limited sample sizes, individuals identifying as Indigenous, Asian, Black or Black African, and Latin American were combined as racial minorities.

c CEGEP: collège d'enseignement général et professionnel.

d A currency exchange rate of CAD $1=US $0.8 was applicable.

### Perceived Effects of Wearable Use on Sleep and Stress Levels

Table 4 presents perceptions about how using wearables affects sleep and stress levels. Overall, 44.3% (n=102) of users reported that using a wearable had a positive effect on their sleep, 4.5% (n=10) reported a negative effect, while 3% (n=7) reported that the effect varied across time. Very similar results were found for the perceived effects of wearable use on stress levels, with 43.6% (n=97) reporting a positive effect, 4.3% (n=10) reporting a negative effect, and 3% (n=7) reporting variations across time.

**Table 4.** Perceived effects of wearable use on sleep and stress levels.

		Values, n (%)
Perceived effects of wearables on sleep		
	Very positive	34 (14.6)
	Positive	37 (16.2)
	Somewhat positive	31 (13.5)
	No impact	111 (48.1)
	Negative	10 (4.5)
	Varied across time	7 (3)
Perceived effects of wearables on stress		
	Very positive	29 (13)
	Positive	46 (20)
	Somewhat positive	22 (9.8)
	No impact	108 (49)
	Negative	10 (4.3)
	Varied across time	7 (3)

### Sleep and Health Care Use in Wearable Users and Nonusers

**Figure 1.**
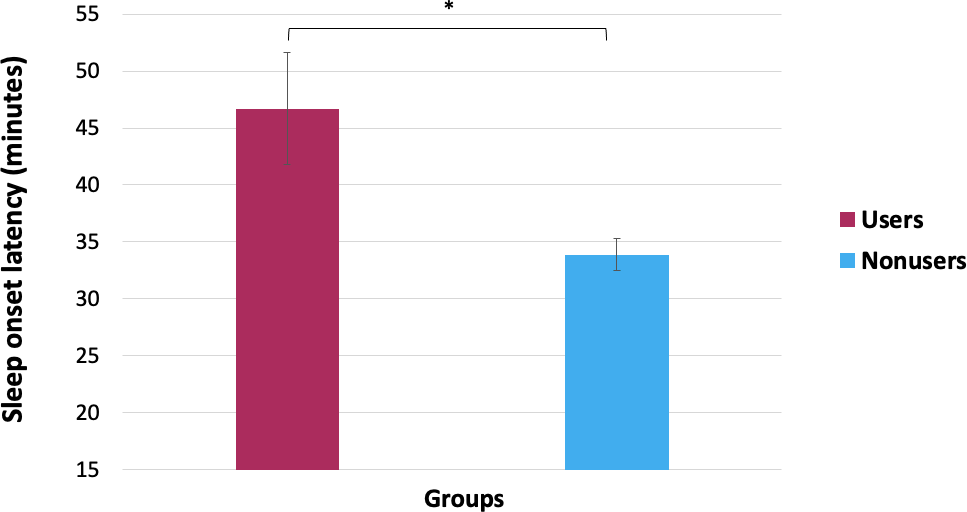
Differences in sleep onset latency between wearable users and nonusers. Error bars represent the standard error of the mean. * *P*<.05.

**Figure 2.**
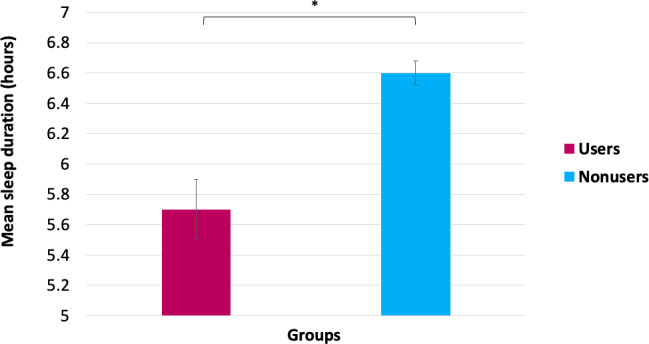
Differences in sleep duration between wearable users and nonusers. Error bars represent the standard error of the mean. * *P*<.05.

**Figure 3.**
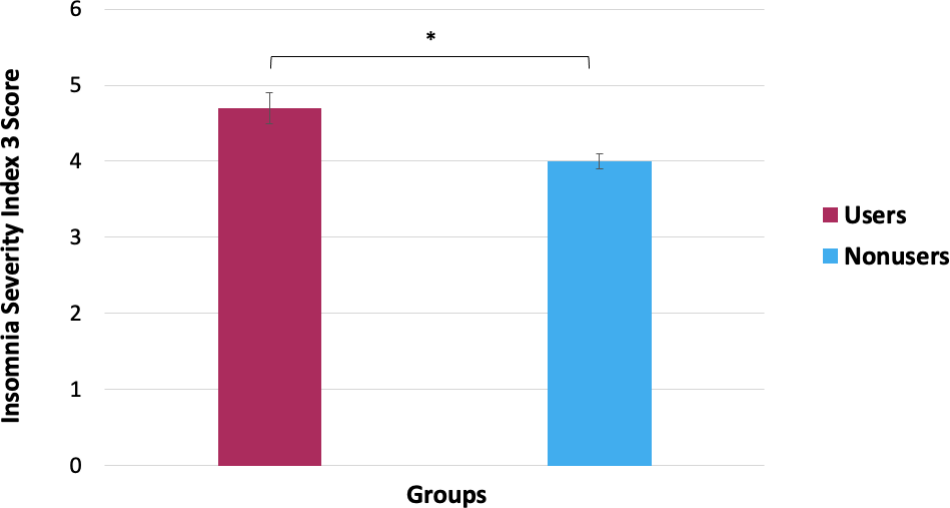
Differences in insomnia severity between wearable users and nonusers. Error bars represent the standard error of the mean. * *P*<.05.

The multivariate covariance analyses indicated a significant effect of wearable use on sleep onset latency, sleep duration, and ISI-3 total scores (*F*_3, 1096_=4.41, *P*=.004; Table 5). Univariate analyses (adjusted for age, sex, ethnicity, occupational status, level of education, income, and insurance coverage) showed that, compared to nonusers, those who had ever worn a wearable had significantly longer sleep latency (*F*_1,1151_=5.21, *P*=.02, ƞp^2^=0.005; Figure 1), shorter sleep duration (*F*_1,1143_=31.60, *P*<.001, ƞp^2^=0.027; Figure 2), and higher insomnia symptoms severity (*F*_1,1119_=4.04, *P*<.05, ƞp^2^=0.004; Figure 3). Only the effect on sleep duration persisted after adjusting for self-reported sleep disorders (*F*_1,1137_=22.39, *P*<.001, ƞp^2^=0.019).

**Figure 4.**
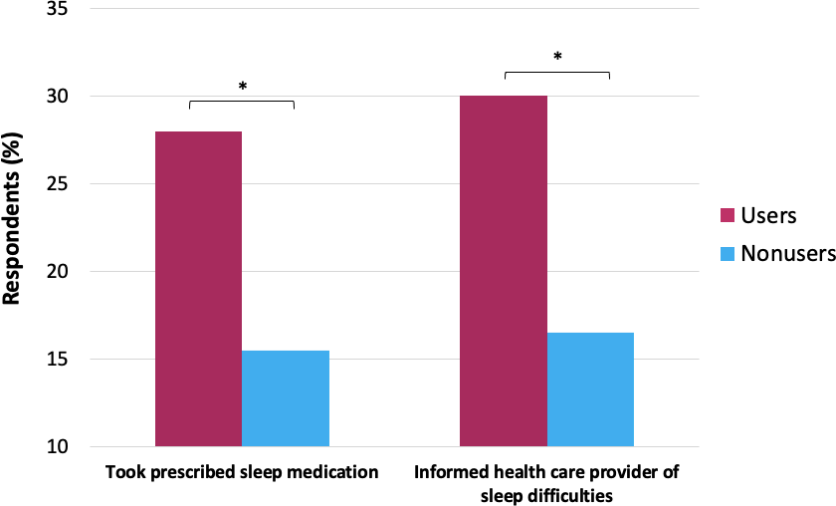
Differences in sleep-related health care between wearable users and nonusers. * *P*<.05.

There were significant associations between the use of wearables and sleep-related health care behaviors, including sleep medication use (*χ*^2^_3_=38.7, *P*<.001) and seeking help from a health care provider for sleep-related problems (*χ*^2^_2_=35.4, *P*<.001; Figure 4). Specifically, the proportion of wearable users was significantly higher in those who reported using prescribed sleep medication (n=85/304, 27.9%) compared to those who did not (n=133/849, 15.7%). The proportion of wearable users was also significantly higher in those who reported having informed a health care provider about their sleep difficulties (n=102/343, 29.7%) compared to those who had not (n=107/654, 16.4%).

**Table 5.** Comparison of sleep in wearable users and nonusers. Univariate analysis of covariates.

	Users, mean (SD)	Nonusers, mean (SD)	*F* test (*df*)	*P* value
SOL^a^ (in minutes)	46.7 (74.5)	33.9 (42.4)	5.2 (1)	.02
TST^b^ (in hours)	5.7 (2.1)	6.6 (1.7)	31.6 (1)	<.001
ISI-3^c^ total score	4.7 (3.0)	4.0 (3.1)	4.0 (1)	.045

a SOL: sleep onset latency.

b TST: total sleep time.

c ISI-3: Insomnia Severity Index-3.

### Moderating Effects of Wearables on Anxiety and Sleep

**Figure 5.**
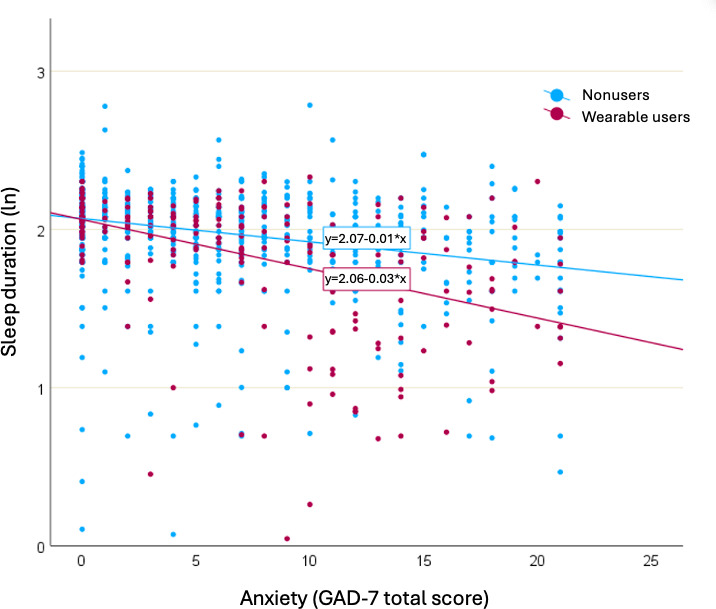
Moderating effect of wearable use on sleep duration and anxiety. ln: natural logarithm. GAD-7: Generalized Anxiety Disorder-7.

In the global sample, there was a statistically significant negative linear relationship between the severity of anxiety symptoms and sleep duration in wearable users (ß=−.014, SE=.002, *P*<.001), and in nonusers (ß=−.167, SE=.186, *P*<.001). However, wearables usage moderated the effect of anxiety symptoms on sleep duration, as evidenced by a statistically significant increase (1.2%) in total variation in sleep duration explained when adding the anxiety*wearables interaction term (*F*_1,1165_=17.5, *P*<.001). As can be seen in Figure 5, wearable users had a steeper decline in sleep duration as anxiety symptoms increased compared to the slope observed in the nonusers. A similar moderation effect was observed in the subgroup without any self-reported sleep diagnosis (n=957), with a weaker but statistically significant increase (0.9%) in total variation in sleep duration explained when adding the anxiety*wearables interaction term (*F*_1,828_=7.8, *P*=.005).

## Discussion

### Principal Results and Implications

This study aimed to investigate the use of sleep wearables in a representative Canadian sample, with a focus on the prevalence, predictors, and perceived effects of using wearables, as well as on the sleep-related differences between users and nonusers. The results from this survey indicate that approximately 1 in 5 Canadians report having used a wearable device to monitor their sleep, which highlights their widespread use in Canada. These numbers parallel those previously observed in Canada and the United States [11,14]. Echoing findings from previous studies, younger individuals and those with a higher income were more likely to report having used a wearable device [11,14]. Sex was not a factor in wearable use. In addition to income level, this study found that being retired as well as being on both provincial and private insurance health care plans were also associated with an increased likelihood of using a sleep wearable. Taken together, these sociodemographic predictors suggest that financial factors limit the accessibility of sleep wearables. Although the price of these devices varies, sleep wearables can be expensive, with many requiring internet access, a compatible smartphone, and subscription fees.

Other sociodemographic characteristics predicting wearable use included having a diagnosed sleep or mental health disorder and being part of a racialized group. Considering the racial disparities that exist in sleep and health [24-26], increased sleep disturbances and limited access to appropriate health care may provide some insight into why racialized individuals may be more likely to turn toward self-monitoring of sleep. Contrary to other studies finding women and those with higher education levels to be more likely to use a wearable [14], sex and education level were not found to predict wearable use in our study.

Among wearable users, nearly half reported positive effects of using wearables for sleep and stress, while a rather small proportion indicated negative effects. While the nature of these subjective benefits remains unclear, one possible explanation could be that some individuals experience positive effects of sleep tracking through increased self-knowledge and awareness [8]. Consistent with the findings of Kuosmanen and Kuosmanen [9], the perception of positive impacts on sleep may be related to wearables enhancing users’ awareness of their sleep patterns, which can motivate healthier sleep behaviors. Although most users perceived positive effects of wearables for sleep and stress, our findings indicate that they also reported worse sleep outcomes, including longer sleep onset latency, shorter total sleep time, and more severe insomnia symptoms than nonusers. Additionally, the proportion of wearable users was nearly twice as high among those who reported taking sleep medications and consulting a health care provider about sleep difficulties than those who reported otherwise. These findings should be interpreted with caution as the timing of the sleep estimation could have been different from the period during which participants had used sleep wearables.

Considering the sociodemographic predictors of wearable use, the presence of sleep and mental disorders may explain the higher rates of sleep disturbances and related health care use found among wearable users. Although this remains to be investigated, it is possible that pre-existing sleep problems may lead individuals to engage in sleep monitoring. It is also possible that some individuals developed sleep issues after they started using wearables. This aligns with concerns raised in the Kuosmanen and Kuosmanen [9] study, where participants reported increased anxiety and stress from continuous sleep tracking, suggesting that, in certain cases, wearables may contribute to worsening sleep difficulties over time. Of note, sleep wearable users in this study experienced a stronger association between anxiety symptoms and sleep duration, providing insights into the potential mechanisms underlying orthosomnia. Although sleep wearables may lead to sleep problems in some individuals, longitudinal studies are yet to be conducted to establish causality.

### Limitations

This study presents several limitations. First, this study’s design was cross-sectional and relied on self-reports, which limits the ability to establish causal relationships between wearable use and factors, such as sleep disturbances and health care–seeking behaviors. While efforts were made to ensure clarity in survey instructions, self-reported data is inherently prone to recall biases, which could impact the reliability and generalizability of the results. Self-report studies are also susceptible to selection bias, which could further reduce external validity. To mitigate this limitation, the sample was semirandomly selected based on age, sex, and geographic region. Future studies could use longitudinal designs, incorporating objective measures of sleep and wearable use. Second, the survey items about wearable use (eg, “Have you ever used a wearable device to monitor your sleep?”) did not assess the timing, frequency, and duration of use. As a result, the moment at which sleep was estimated as part of this survey may not always match the period when wearable devices were used. As such, an important next step will be to study current wearable users and gather detailed information on usage patterns, including frequency, duration, and consistency of use. Furthermore, other important factors such as device type and comfort, motivation for use, reasons for discontinuing use, and pairing of devices to mobile apps remain to be assessed to better contextualize perspectives on wearables. Third, the proportion of the sample reporting the use of wearables was relatively small (n=231), and the sample had a limited representation of racial minorities, restricting the ability to draw conclusions that are broadly applicable to diverse populations. Fourth, it is possible that wearable users based their responses to sleep-related items, such as those in the PSQI, on data summaries provided by their devices rather than on their subjective estimates of sleep. As such, observed differences in “subjective” sleep ratings between groups may be confounded by the source of information used. Future studies should clarify in survey instructions when responses should reflect subjective estimates rather than information obtained from devices. Lastly, the survey was conducted in 2021. Given the rapid advancements and evolving features of wearable technologies, the findings presented in this study may no longer fully reflect the current landscape. Additionally, the survey responses may have been influenced by the context of the COVID-19 pandemic, a period marked by increased stress, sleep disturbances, symptoms of anxiety and depression, and overall declines in psychological well-being at the population level [27,28]

### Future Directions

Additional studies are needed to expand our understanding of how orthosomnia develops and is maintained. Future studies are also needed to better understand how sleep misperception, especially in people with sleep disorders such as insomnia, influences wearables use and orthosomnia. Identifying individual characteristics linked to harmful use of wearables could help direct preventive targeted interventions. Future studies should also investigate why consumers perceive wearables as beneficial or harmful to sleep and stress, and explore the factors that contribute to the variability of wearables’ effects over time. Moreover, research initiatives are needed to examine the multidisciplinary implications of using wearables in clinical interventions. There is a need to understand the effects of wearable use in diverse clinical populations. As highlighted by Baron et al [10], collaborating with patients to incorporate wearable devices into treatment plans may enhance communication between patients and providers and alleviate patient burden. Future research should also consider financial constraints affecting the accessibility of these sleep wearables and prioritize the recruitment of individuals with more diverse racial backgrounds.

### Conclusions

The present results from a national survey highlight the widespread use of wearable technology in Canada and some of the sociodemographic factors linked to a higher likelihood of use, including sleep and mental disorders. This study also emphasizes their perceived positive effects, in contrast to the sleep and mental health data that supported a more nuanced relationship between sleep, mental well-being, and wearables. Importantly, this study stresses the need for objective evaluation of the benefits and potential drawbacks of using wearable devices to monitor sleep at the individual level. More research is required to better understand individual profiles, device characteristics, and context of use that could be linked to beneficial versus harmful use of wearables. Importantly, there is a pressing need to develop frameworks for the clinical use of sleep wearables, considering individual characteristics that may impact outcomes, such as anxiety symptoms, unrealistic expectations, poor sleep practices, or sleep state misperceptions, keeping in mind that the sociodemographic predictors studied only account for 15.8% of the variance, thus necessitating a consideration of other factors.

## Supplementary material

10.2196/68816 Checklist 1 CHERRIES checklist. CHERRIES: Checklist for Reporting Results of Internet E-Surveys.

## Abbreviations

CEGEP: collège d'enseignement général et professionnel
GAD-7: Generalized Anxiety Disorder-7
ISI-3: Insomnia Severity Index-3
PSQI: Pittsburgh Sleep Quality Index
